# The impact of RASopathy-associated mutations on CNS development in mice and humans

**DOI:** 10.1186/s13041-019-0517-5

**Published:** 2019-11-21

**Authors:** Minkyung Kang, Yong-Seok Lee

**Affiliations:** 1 Department of Physiology, Seoul National University College of Medicine, 103 Daehak-ro, Jongro-gu, Seoul, 03080 South Korea; 2 Department of Biomedical Sciences, Seoul National University College of Medicine, Seoul, 03080 Korea; 3 Neuroscience Research Institute, Seoul National University College of Medicine, 103 Daehak-ro, Jongro-gu, Seoul, 03080 South Korea

**Keywords:** RAS, MAPK, neurodevelopmental disorders, cognition, mutant strains mouse

## Abstract

The RAS signaling pathway is involved in the regulation of developmental processes, including cell growth, proliferation, and differentiation, in the central nervous system (CNS). Germline mutations in the RAS signaling pathway genes are associated with a group of neurodevelopmental disorders, collectively called RASopathy, which includes neurofibromatosis type 1, Noonan syndrome, cardio-facio-cutaneous syndrome, and Costello syndrome. Most mutations associated with RASopathies increase the activity of the RAS-ERK signaling pathway, and therefore, most individuals with RASopathies share common phenotypes, such as a short stature, heart defects, facial abnormalities, and cognitive impairments, which are often accompanied by abnormal CNS development. Recent studies using mouse models of RASopathies demonstrated that particular mutations associated with each disorder disrupt CNS development in a mutation-specific manner. Here, we reviewed the recent literatures that investigated the developmental role of RASopathy-associated mutations using mutant mice, which provided insights into the specific contribution of RAS-ERK signaling molecules to CNS development and the subsequent impact on cognitive function in adult mice.

## Introduction

**Fig. 1:**
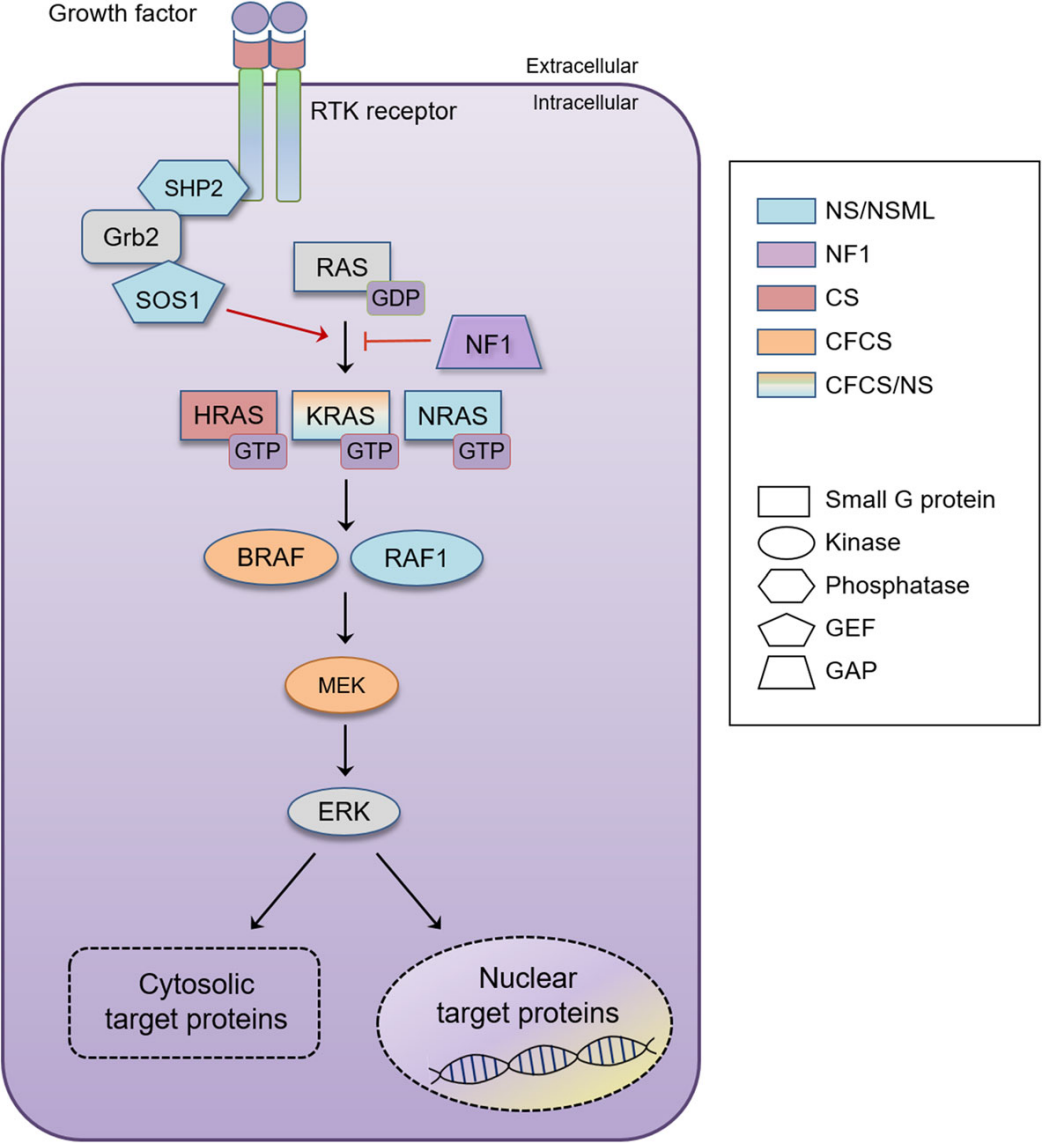
The RAS-ERK signaling pathway and associated disorders. A simplified RAS-ERK signaling pathway. Genes frequently mutated in RASopathy are colored based on the RASopathy and are displayed as a polygon depending on their functional categories. NS/NSML, Noonan syndrome/Noonan syndrome with multiple lentigines; NF1, Neurofibromatosis type 1; CS, Costello syndrome; CFCS, Cardio-facio-cutaneous syndrome; GEF, guanine exchange factor; GAP, GTPase activating protein.

The RAS-extracellular signal-regulated kinase (ERK) pathway is a highly conserved signaling cascade that transduces signals from membrane receptors to the cytoplasm and nucleus by protein–protein interactions and phosphorylation [1–3]. It plays a critical role in controlling various cellular processes, including cell growth, survival, proliferation, and differentiation, in developing and adult tissues, such as the brain [2, 4]. *RAS,* which is composed of a multigene family that includes *HRAS*, *KRAS*, and *NRAS,* encodes a small guanosine nucleotide-bound GTPase protein, and the activation of the RAS-ERK signal transduction is initiated by the binding of growth factors to G-protein-coupled receptors, such as receptor tyrosine kinases (RTKs) and cytokine receptors. *RAS* is activated by guanine nucleotide exchange factors (GEFs), such as SOS1, whose activity is regulated by multiple adaptor proteins, including GAB1 and GRB2 (Fig. 1) [5]. On the contrary, GTPase activating proteins (GAPs), such as NF1, switch RAS activity off by hydrolyzing GTP to GDP. The GTP-bound form of active RAS leads to the activation of its direct downstream effector, RAF. *RAF* encodes a serine/threonine kinase and represents the RAF family, which also includes ARAF, BRAF, and RAF1. RAF phosphorylates and activates the MAPK kinase, MAPK/ERK kinase 1/2 (MEK1/2), which in turn activates ERK1 and ERK2 by phosphorylating the tyrosine and threonine residues on ERK1/2 [6]. ERK1 and ERK2 are homologous subtypes of the ERK family and are final effectors of the RAS-ERK pathway. ERK1/2 affect a large number of downstream molecules, such as nuclear components, transcription factors, and membrane proteins [7].

Since the RAS-ERK pathway is critically involved in multiple biological processes, germline mutations in RAS-ERK signaling components can cause a class of developmental disorders that are collectively called RASopathy [3, 8, 9]. RASopathy affects approximately 1 in 1,000 live births worldwide and shares a common molecular mechanism, such as mutations in RAS-ERK signaling components [4]. Representatively, RASopathy includes 1) neurofibromatosis type 1, which is caused by loss of function mutations in *NF1*; 2) Noonan syndrome, caused by gain of function mutations in *PTPN11*, *SOS1*, *SHOC2*, *CBL*, *KRAS*, *NRAS*, *BRAF*, *RAF1*, and *MEK1*; 3) Noonan syndrome with multiple lentigines that is caused by mutations in *PTPN11* and *RAF1*; 4) cardio-facio-cutaneous syndrome, which is caused by either gain of function or loss of function mutations in *BRAF*, *KRAS*, *MEK1*, and *MEK2*; 5) Costello syndrome, caused by gain of function mutations in *HRAS*; and 6) neurofibromatosis type 1-like syndrome (NFLS or Legius syndrome) that is also caused by loss of function mutations in *NF1*. RASopathies share typical characteristics, such as a short stature, craniofacial dysmorphism, cardiac defects, and neurocognitive impairments that are accompanied by abnormal brain development [10]. However, each RASopathy also displays distinct and unique symptoms, depending on the mutated genes [3, 11]. Consistently, recent studies using mouse models of RASopathies have demonstrated that each disorder also shows disease-specific abnormalities in central nervous system (CNS) development. Here, we review the distinctive roles of RAS-ERK signaling molecules in CNS development that were revealed by investigating the deficits in CNS development of RASopathies (Tables 1 and 2). Furthermore, we also review how RASopathy-associated mutations affect cognitive function in mice and human.

**Table 1:** Human patients with RASopathies and their phenotypes

Disease	Associated genes	CNS structural phenotypes	Other phenotypes
Neurofibromatosis type 1	*NF1* (95%) [12]	Neurofibromas , abnormal cortical development [13], abnormal glial development [14], macrocephaly	Below-average IQ, ADHD, impaired executive functioning, deficits in visual-spatial skills [15, 16], hyperpigmentation of melanocytes, hamartomas of the iris [17, 18], bone malformation, cardiac defects [19, 20]
Noonan syndrome, Noonan syndrome with multiple lentigines	*PTPN11* (>50%) [21]*, RAF1* (3-17%) [22, 23]*, SOS1* (9-13%) [24] *KRAS* (<2%) [25, 26], *BRAF* (<2%) [22], *MEK1/2* (<2%) [27]	Cerebellar ectopia [28, 29], temporal lobe anomaly, hydrocephalus, cerebral abscess [30–32], epilepsy, cortical dysplasia [33]	Neurocognitive delay [33–35], typical facial abnormalities, short stature, motor delay, increased risk of cancer, cardiac defects [34–40]
Cardio-facio-cutaneous syndrome	*BRAF* (43-78%) [41–43], *MEK*1/2 (7-11%) [42, 43], *KRAS* (5-8%) [25, 43]	Ventriculomegaly, hydrocephalus [44–50], atrophy [44, 46, 51–54], migration and myelination abnormalities, agenesis of corpus callosum [50, 52, 55–57]	Neurological abnormalities, seizures, tactile defensiveness, learning disabilities [4, 50, 55], craniofacial defects, cardiac defects [4, 58, 59], motor delay, hypotonia [4, 50, 55]
Costello syndrome	*HRAS (85-90%)* [60–62], *KRAS* (7%) [63], *BRAF* (4-6%) [27], *MEK*1/2 (2-3%) [27]	Ventricular abnormalities [64–67], cerebral malformations [64, 65, 67–71], cerebellar abnormalities [66, 69, 71–74], macrocephaly [59, 60]	Mental retardation [59, 60], facial features, loose skin, severe failure to thrive, predisposition to tumors [59, 60]

*IQ* Intelligence quotient, *ADHD* Attention deficit hyperactivity disorder;

**Table 2:** RASopathy mouse models and their phenotypes

Gene	Mouse model	CNS structural phenotypes	Other phenotypes
*Nf1*	*Nf1* homozygous knockout	Defects in the neural tube, hyperplasia of neural crest-derived ganglia [75]	Embryonic lethality, heart defects, delay in organ development [76, 77]
	*Nf1* heterozygous knockout	Increased number of astrocytes [78, 79]	Impaired synaptic plasticity, impaired spatial learning, heart defects [80–82]
	Synapsin 1-dependent *Nf1* ablation	Reduced size and weight of the forebrain, reduced cortical thickness, increased astrogliogenesis [83]	Learning deficits, growth retardation [83, 84]
	hGFAP-dependent *Nf1* ablation	Increased gliogenesis, enlarged cerebral cortex, defective GNP migration and proliferation [85–88]	Postnatal lethality, growth retardation [86, 87]
	BLBP-dependent *Nf1* ablation	Increased glial lineage proliferation, abnormal neuronal differentiation [89]	Postnatal lethality [89]
	Nestin-dependent *Nf1* ablation (induction in adulthood)	Unlocked latent oligodendrocyte lineage, defective GNP proliferation and migration, increased adult hippocampal neurogenesis [88, 90, 91]	Spontaneous antidepressive-like behavior [91]
*Ptpn11*	Nestin-dependent *Ptpn11* ablation	Decreased neural stem cell proliferation, lamination defects, reduced number of neurons, increased number of astrocytes [92]	Postnatal lethality, growth retardation [92]
	Olig1-dependent *Ptpn11* ablation	Decreased number of oligodendrocyte precursors and mature oligodendrocytes, reduced axonal myelination [93]	Developmental abnormalities [93]
	Olig2-dependent *Ptpn11* ablation	Decreased number of oligodendrocyte precursors, hypomyelination [94]	Postnatal lethality, severe shivering [94]
	Olig2-dependent *Ptpn11*^Q79R^ knock-in	Increased number of oligodendrocyte precursors, abnormal myelination [94]	Not described
	Nestin-dependent *Ptpn11*^E76K^ knock-in	Hydrocephalus, aberrant development of ependymal cells, reduced proliferation, enhanced glial differentiation [95]	Postnatal lethality, dome-shaped head, reduced anxiety behavior, hyperactivity, impaired motor function [95]
	*Ptpn11*^D61G^ herozygous knock-in	Increased neurogenesis, decreased gliogenesis [96]	Impaired synaptic plasticity, impaired spatial learning, short stature, craniofacial dysmorphia [97, 98]
*Kras*	Synapsin1-dependent *Kras*^G12V^ knock-in	Enhanced GABAergic synaptogenesis [99]	Increased inhibitory tone, impaired spatial learning [99]
*Hras*	*Hras*^G12V^ homozygous knock-in	Hypertrophy of the brain and pyramidal neurons [100]	Impaired spatial learning, facial dysmorphia, cardiac defects [100]
	aCaMKII-dependent *Hras*^G12V^ knock-in	Increase in docked vesicles [101]	Increased synaptic plasticity, enhanced spatial learning [101]
*Braf*	Nestin-dependent *Braf* ablation	Impaired neuronal differentiation, dysmyelination, defective oligodendrocyte differentiation [102, 103]	Postnatal lethality, growth retardation, defective motor coordination, neuromuscular defects [102, 103]
	*Braf*^V600E^ heterozygous knock-in	Increased number of GFAP positive cells in the DG [104]	Reduced life span, growth retardation, facial dysmorphia, cardiomegaly, epileptic seizures [104]
*Raf1*	*Raf1* heterozygous knockout	Small granule cell volume, increased cell death, reduced neuronal maturation [105]	Postnatal lethality, growth retardation, apoptosis in the lung and liver, limbs coordination problems [106]
	*Raf1*^L613V^ heterozygous knock-in	Increased density of astrocytes, enhanced OPCs density [107]	Enhanced learning and memory [107]
*Mek1/2*	*Mek1*^Y130C^ homozygous knock-in	Increased astrocyte density, increased number of cortical oligodendrocytes [108]	Pulmonary artery stenosis, cranial dysmorphia [108]
	Nestin-dependent *Mek1/2* ablation	Decrease of astrocyte precursors and OPCs, failure of gliogenesis [109]	Early postnatal lethality [109]
	hGFAP-dependent *Mek1/2* ablation	Suppressed generation of astrocyte precursors and OPCs, failure of gliogenesis [109]	Postnatal lethality [109]
	hGFAP-dependent *Mek1*^S218E,S222E^	Increase in astrocyte precursors and mature astrocytes, reduction of neuron number [109]	Not described

*hGFAP* Human glial fibrillary acidic protein, *BLBP* Brain lipid binding protein, *GNP* Granule neuron progenitor, *DG* Dentate gyrus, *OPCs* Oligodendrocyte progenitor cells

### RAS-ERK signaling and nervous system development

The RAS-ERK signaling pathway is tightly regulated during CNS development and many studies have demonstrated that the dysregulation of this signaling pathway results in aberrant brain development. There are a number of studies demonstrating that ERK1/2, the final effectors of RAS-ERK signaling, are involved in cell proliferation and differentiation in the nervous system [110]. Activation of ERK signaling is required for neural stem cells (NSCs) to maintain their ability to self-renew and form neurospheres, indicating that ERK may act as a critical regulator in the maintenance of NSCs [111]. In addition, it has also been shown that ERK signaling promotes neuronal survival by multiple mechanisms [112, 113]. For example, an ERK-activated kinase, ribosomal S6 Kinase (RSK), phosphorylates the pro-apoptotic protein BAD and suppresses BAD-mediated apoptosis in neurons [112]. ERK was also shown to regulate the activation of anti-apoptotic regulators, such as Bcl-2, CREB, and STAT3/5, and subsequently promote cell survival [112, 114, 115]. However, in spite of the crucial role of ERK in neuronal survival, aberrant and long-lasting ERK activation has also been implicated in neurodegenerative diseases [116, 117].

Several studies have implied that the MEK/ERK signaling cascade has a crucial role in neurogenesis. ERK2 is necessary for regulating the proliferation of neurogenic precursors and the positive regulation of neurotrophin-induced neurogenesis by the MEK-C/EBP pathway during cortical development [118, 119]. Despite the evidence that MEK is required for neurogenesis, in vivo and in vitro studies have demonstrated that ERK also regulates and maintains the pool of glial populations in the developing brain [109]. NSC-specific ablation of *Mek1/2* induces a complete blockade of glial specificity and gliogenesis failure, while *Mek1* gain of function promotes precocious glial progenitor specification in mice [109]. *S*everal studies have demonstrated that in vitro, *Erk1* and *Erk2* are critical components of proliferation in cultured rat astrocytes, and that MEK/ERK signaling induces gliogenic signals, such as SDF-1a and FGF2 [120–122]. Consistently, treatment with the MEK inhibitor PD98059 induced a reduction in astrocytic growth, suggesting that MEK/ERK signaling is involved in astrocyte proliferation [122]. In addition, the chemical inhibition of MEK also impairs the ability of oligodendrocyte precursors to differentiate into mature oligodendrocyte in vitro, suggesting that both oligodendrocytes and astrocytes are regulated by ERK signaling [103]. Several studies demonstrated that the pharmacological inhibition of ERK1/2 signaling in oligodendrocyte progenitors negatively regulates differentiation and the transition of early progenitors to late oligodendrocyte progenitors [123–125]. Furthermore, ERK signaling promotes oligodendrocyte myelination [126]. However, there are conflicting results about the role of ERK signaling in the differentiation of oligodendrocyte progenitors into mature oligodendrocytes. Recently, Suo and colleagues demonstrated that MEK inhibitors significantly enhance the differentiation of oligodendrocyte precursor cells into oligodendrocytes in vitro and in vivo [127]. Consistently, many studies have suggested that increased ERK activity negatively regulates oligodendrocyte differentiation. For example, ERK1/2 activation, which is induced by high dose stimulation of neuregulin-1 or fibroblast growth factor-2 in mature oligodendrocytes, results in downregulated myelin proteins and aberrant cell cycle re-entry [128–130].

The RAS-ERK signaling pathway also regulates the expression of transcription factors, such as cell fate determinants. Numerous studies demonstrated that the enhanced activity of RAS-ERK signaling induces the expression of the transcription factor *OLIG2,* which promotes the fate of NSCs to the glial lineage [85, 90, 108]. Furthermore, the activation of RAS-ERK signaling promotes the expression of the pro-neural gene *Achaete scute-like 1* (*Ascl1*) but blocks pro-neural gene *Neurogenin 2* (*Neurog2*) expression. *Neurog2* specifies glutamatergic neuronal cell fate in dorsal progenitors, while *Ascl1* specifies neocortical gamma-aminobutyric acidergic (GABAergic) neurons and oligodendrocyte precursor cells [131–133]. Therefore, during normal early developmental stages, RAS-ERK signaling activity is kept low so that *Neurog2* is able to promote glutamatergic neuronal differentiation of embryonic cortical progenitors. However, in an abnormal context where the RAS-ERK signaling is elevated, *Neurog2* expression is switched to *Ascl1* expression [134]. During moderate activation of RAS-ERK signaling, *Ascl1* expression promotes GABAergic neuronal differentiation, while *Ascl1* promotes proliferative glioblast phenotypes when RAS-ERK signaling is highly active [134].

RAS interacts with and regulates other signaling pathways in addition to the MEK/ERK cascade. As one of the main effector pathways of RAS, the phosphatidylinositol 3-kinase (PI3K)-AKT pathway regulates protein synthesis and variety of cellular processes such as cell growth, cycle entry, and cellular survival [135–137]. The Ras and PI3K-AKT pathway were shown to activate and inhibit each other via multiple cross-talks [138]. Studies using rodent models have reported distinct phenotypes and revealed a pivotal role of PI3K signaling in nervous systems. For instance, deleting a PI3K isoform PI3Kγ in mice impaired synaptic plasticity and behavioral flexibility, while its overexpression through viral vector resulted in impaired synaptic plasticity and spatial learning [139, 140]. The Janus kinase (JAK)-signal transducer and activator of transcription (STAT) pathway is also a well characterized cascade known to interact with RAS-ERK [141]. JAK activation stimulates cell proliferation, differentiation, cell migration and apoptosis, and there are compelling evidences that JAK-STAT pathway plays essential roles in synaptic plasticity [142].

### RASopathies and central nervous system development

#### Neurofibromatosis type 1

Neurofibromatosis type 1 (NF1) is a relatively common developmental disease that affects 1 in 3,000 individuals and is diagnosed by both somatic and behavioral symptoms [20, 143]. NF1 is caused by loss of function mutations in *NF1* alleles [10, 143, 144]. The *NF1* gene encodes a GAP for RAS, neurofibromin, which promotes the conversion of active RAS-GTP to inactive RAS-GDP, thus, negatively regulating the RAS-ERK signaling pathway [145, 146]. Therefore, loss of function mutations in *NF1* result in the hyperactivation of RAS-ERK signaling. As mutations in the *NF1* gene lead to abnormal cell growth, proliferation, and differentiation, individuals with NF1 frequently display neurofibromas, hyperpigmentation of melanocytes, and hamartomas of the iris [17, 18]. Additionally, common features of NF1 include bone malformations, cardiac defects, and neurocognitive impairments [19, 20]. More than 75% of NF1 patients suffer from cognitive deficits, such as below-average IQ and specific deficits in attention, executive functioning, and visual-spatial skills [15, 16].

Although tumor development in the peripheral nervous system is a hallmark of NF1, a variety of CNS abnormalities, including neurofibroma, have been reported in NF1 patients [147]. For example, abnormal cortical lamination and a compressed cerebral cortex were observed in the brains of NF1 patients, indicating a critical role for *NF1* in cortical development [13]. Interestingly, several studies have also suggested that NF1 is associated with deficits in glial development. For example, children with NF1 display abnormalities in astrocyte growth regulation and tend to develop astrocytoma [14, 148]. Similarly, a postmortem study reported that three NF1 brains exhibited extensively increased astrogliogenesis [149]. Specifically, an association between an enlarged corpus callosum and severe learning disabilities in a subpopulation of NF1 patients has been reported [150, 151]. Moore and colleagues also reported that the total brain volume, especially the gray matter, was significantly larger in NF1 subjects than in children and adolescents without NF1. The gray matter volume in NF1 subjects was inversely correlated with their degree of learning disability [150]. Taken together, individuals with NF1 display CNS developmental abnormalities, including promoted astrogliogenesis and structural malformation, which might be associated with learning disabilities.

*Nf1* homozygous knockout mice (*Nf1*^-/-^) die *in utero* because of severe heart malformations, a delay in renal, hepatic, and skeletal muscle development, and hyperplasia of neural crest-derived sympathetic ganglia [76, 77]. In addition, *Nf1*-deficient mouse embryos exhibit defects in the neural tube, including exencephaly or the thinning of the dorsal telencephalic wall, although the targeted allele in this study was slightly different from previous investigations [75]. Therefore, a heterozygous knockout mouse line (*Nf1*^+/-^) has been extensively used to investigate the cellular mechanisms underlying NF1 etiology [80, 81, 83, 84, 152, 153]. Silva and colleagues showed that *Nf1*^+/-^ mice display impaired spatial learning and impaired hippocampal synaptic plasticity [80, 81]. Mechanisms underlying the deficits in learning and synaptic plasticity in NF1 mouse models have been extensively reviewed in previous publications [8, 154]. In line with human patients, *Nf1* heterozygous mutant mice showed developmental abnormalities in the heart and neural crest-derived tissues, and an increased number of astrocytes with high levels of glial fibrillary acidic protein (GFAP) in the periaqueductal grey, nucleus accumbens, and hippocampus [76, 79].

Ablation of *Nf1* only in neurons by using the Synapsin I promoter (*Nf1*^Syn1^) led to growth retardation, including reduced body weight and size, that was sustained into adulthood [83]. *Nf1*^Syn1^ conditional knockout (CKO) mice exhibited reduced size and weight of the forebrain, but not other brain regions [83]. Histological analyses of CKO mice also revealed remarkable defects in the cerebral cortex, such as a reduction in cortical thickness [83]. Neuronal loss in mutant cortices was not detected; however, interestingly, CKO mice displayed extensive GFAP immunoreactivity throughout the cerebral cortex, hippocampus, and brainstem, which indicates increased astrogliogenesis [83]. These results indicate that *Nf1* has an indispensable role in CNS development, and that *Nf1*-deficient neurons induce astroglial hypertrophy and GFAP induction through a paracrine effect [83, 155].

Several studies suggested that neurofibromin might be required for NSCs or neuroglial progenitor function, and that *Nf1* mutations affect both astroglial and neuronal lineages. Studies using a well-characterized human GFAP (hGFAP)-Cre transgenic mouse line have demonstrated that *Nf1* plays a critical role in CNS development. Typically, hGFAP-Cre expression is first detected in radial glia, which give rise to both neuronal and glial lineage cells, around embryonic day 13 [156]. Mutant *Nf1*^hGFAP^ CKO mice, which lack neurofibromin in the majority of their cortical neurons and astrocytes, were born in normal numbers, but became noticeably smaller than their littermates over time, and typically died by four months of age [86, 87]. *Nf1*^hGFAP^ CKO mice displayed enlarged cerebral cortices and an increased brain to body weight ratio caused by the enlarged cortex [85, 88]. The mutant mice also exhibited a notably smaller cerebellum, compared with littermates, and defective migration and proliferation of granule neuron progenitors [88]. In addition, *Nf1*^hGFAP^ CKO mice failed to form cortical barrels in the somatosensory cortex, although segregation of thalamic axons within the somatosensory cortex was unaffected [87]. Consistent with NF1 patients, the mutant mice displayed increased GFAP-positive astrocytes throughout both the gray and the white matter, including the corpus callosum and anterior commissure [86]. Wang and colleagues also showed that the *Nf1*^hGFAP^ CKO mice display increased gliogenesis at the expense of neurogenesis in the neonatal period and during adulthood [85]. Due to the altered ratio of glia to neurons, *Nf1*^hGFAP^ CKO mice displayed a smaller olfactory bulb and an enlarged corpus callosum, providing a link between brain structural abnormalities and cognitive impairments in animal models and those seen in NF1 patients [85]. Similarly, *Nf1* inactivation in neuroglial progenitors using a brain lipid binding protein (BLBP)-Cre mouse strain also led to increased glial proliferation and abnormal neuronal differentiation in vivo [89]. However, it is also noteworthy to mention that deleting *Nf1* using GFAP-Cre did not impair either learning or synaptic plasticity in adult mice [84].

Recent studies reported that *Nf1* regulates cell fate specificity and cellular processes in both the developmental stage and in adulthood. Inactivation of *Nf1* in adult NSCs unlocked a latent oligodendrocyte lineage and allowed NSCs to produce all three lineages in vivo [90]. Similarly, postnatal *Nf1* ablation using Nestin-CreERT2 was sufficient to cause cerebellar abnormalities, including defective cerebellar foliation, granule neuron progenitors (GNPs) proliferation, and migration [88]. Also, deletion of *Nf1* in adult hippocampal neural progenitor cells led to enhanced proliferation and an increase in new neurons in the dentate gyrus [91].

Since *Nf1* also functions as a tumor suppressor gene, in vitro studies in various cell types have suggested that *Nf1* mutations are associated with growth abnormalities, such as increased proliferation of oligodendrocyte precursors in the embryonic spinal cord [157] and Schwann cells [158]. Particularly, *Nf1*^-/-^ and *Nf1*^+/-^ NSCs generate increased numbers of morphologically abnormal, immature astroglial cells in vitro [159]. The increase in astroglial progenitors and proliferating cells seen in vitro was also observed in *Nf1*^-/-^ and *Nf1*^+/-^ embryonic brains and *Nf1*^+/-^ adult brains in vivo [159]. In addition, Lee and colleagues showed that *Nf1*^-/-^ NSCs from the brainstem exhibit increased proliferation and glial cell differentiation in vitro and in vivo; however, the lack of effect on neocortex NSCs proliferation or gliogenesis suggests that the effects of *Nf1* gene inactivation are brain region-specific [160].

What would be an underlying mechanism for the enhanced glial population in NF1? It has been demonstrated that *Nf1* inactivation in neural stem/progenitor cells can alter glia/neuron fate specification by promoting the expression of *Olig2*, a basic-helix-loop-helix transcription factor that is required for oligodendrocyte progenitor cell specification [161]. *Nf1*^hGFAP^ CKO and *Nf1*^BLBP^ CKO mutant mice showed increased *Olig2* expression, suggesting that *Nf1* suppresses *Olig2* expression and the oligodendrocyte progenitor lineage in neonatal subventricular zone progenitor cells [85, 160]. In concordance with the neonatal study, inactivation of *Nf1* in adult NSCs also resulted in increased *Olig2* expression [90]. In conclusion, these studies with *Nf1* mutant mice revealed the essential role of NF1 in CNS development, including the gross morphology and proper formation of several brain region structures, and the regulation of cell fate.

Along with structural abnormalities in CNS, several lines of evidence suggest that the distribution of *NF1* in single neuronal cell type may also contribute to cognitive deficits in NF1. Transcriptome analyses of mouse brain have unveiled the enriched *NF1* expression in inhibitory neurons rather than the in excitatory neurons, and provided a clue as to how *NF1* mainly carries out its role in inhibitory synaptic function [162]. Furthermore, based on the conserved expression pattern of *NF1* in human brain, it is suggested that the enriched expression of *NF1* in inhibitory neurons may underlie cell type-specific pathophysiology and cognitive deficits in NF1 [163].

*Nf1* mutant mice mimic most of the CNS features found in NF1 human patients, including increased brain volume, enlarged corpus callosum and cortical area, and especially, enhanced gliogenesis, which may be closely associated with structural abnormalities. Despite compelling evidences of the expression of glial lineage transcription factors such as *Olig2* increasing as RAS-ERK highly activates [85, 90, 108], yet it is unclear how RAS-ERK pathway regulates cell fate determinants. Thus, for understanding CNS abnormalities in NF1 patients, it is worth investigating the expression regulations of cell fate determinants with regard to RAS-ERK activity.

#### Noonan syndrome and Noonan syndrome with multiple lentigines

Noonan syndrome (NS) is an autosomal dominant genetic disorder with an incidence of 1 in 2,500 live births [31, 164, 165]. This complex disorder occurs both in familial and sporadic forms [166]. Germline mutations in genes involved in RAS-ERK signaling pathway have been reported to be associated with NS, such as the gain of function mutations in *protein tyrosine phosphatase non-receptor type 11* (*PTPN11*), *son of sevenless homolog 1* (*SOS1*), *Kirsten rat sarcoma viral oncogene homolog* (*KRAS*), *neuroblastoma RAS viral oncogene homolog* (*NRAS*), *Raf-1 proto-oncogene* (*RAF1*), *BRAF*, *soc-2 suppressor of clear homolog* (*SHOC2*), and *MEK1*, and the loss of function mutations in *Cbl proto-oncogene* (*CBL*) [25, 63, 167]. Above all, mutations in *PTPN11,* which encodes the non-receptor protein phosphatase SHP2, account for approximately 50% of NS cases [167]. Patients with NS are characterized by typical facial abnormalities, such as a broad forehead, sparse eyebrows, a low-set and posteriorly rotated ear, and a webbed neck, while other important features include a short stature, motor delay, increased risk of cancer, and cardiac defects [34–40]. Noonan syndrome with multiple lentigines (NSML) patients have most of the clinical symptoms observed in individuals with NS, but they also display increased penetrance of hypertrophic cardiomyopathy and lentigines [168]. Distinct from NS, *PTPN11* loss of function mutations result in NSML [168].

Between 30%-50% of NS patients show a variable degree of neurocognitive delay, but there are relatively few reports of CNS malformations in NS individuals [34, 35]. Two cases of NS were reported to be associated with cerebellar ectopia [28, 29]. In addition, there are several reports of NS being associated with a temporal lobe anomaly, hydrocephalus, cerebral abscess, and malignant Schwannoma [30–32]. In particular, Saito and colleagues reported one case of an NS patient with severe mental retardation and intractable epilepsy [33]. The patient also displayed cortical dysplasia, including dilated perivascular spaces and a dysplastic lesion in the left temporal lobe [33].

Mutant mice harboring NS-associated *Sos1*^E846K^, *Kras*^V14I^, and *Raf1*^L613V^ displayed a short stature, facial dysmorphia, growth retardation, and cardiac defects, which are characteristic features of NS patients [169–172]. Since *PTPN11* mutations are the majority among NS cases, Shp2 mutant mice are one of the most studied models of NS [96–98, 173, 174]. A subpopulation of NS patients have a constitutively active mutation Shp2^D61G^, which has a highly increased phosphatase activity [175, 176]. The homozygous Shp2^D61G^ mutation was eventually embryonically lethal, as the embryos were grossly hemorrhagic and edematous, showed a decreased liver size, and had cardiac defects [98]. However, half of heterozygous Shp2^D61G^ mice that carried only one copy of the mutant allele (Shp2^D61G/+^) survived, and displayed a short stature and craniofacial dysmorphia, such as wide-set eyes, a broad forehead, and a triangular face, which were similar to NS patients [98]. Heterozygous Shp2^D61G^ mice also showed deficits in spatial learning and memory and had impaired synaptic plasticity [97]. Mice carrying a milder mutation, Shp2^N308D^, displayed some cardiac defects and mild impairment to spatial learning and memory that was consistent with human cases [97, 98]. Neural crest cell-specific Shp2^Q79R^ resulted in craniofacial defects and growth retardation [170]. Neural stem cell-specific expression of Shp2^E76K^ by using Nestin-Cre resulted in hydrocephalus due to aberrant development of ependymal cells [95]. In addition, Shp2^E76K^-expressing mice showed hyperactivity accompanied by reduced anxiety behavior, and impaired motor function [95]. Global Shp2^D61Y^ expression resulted in embryonic lethality, while epiblast-specific Shp2^D61Y^ expression induced embryonic cardiac defects [173].

SHP2 is a growth factor-regulated phosphatase that modulates both the RAS-ERK and the gp130-JAK-STAT pathways [177, 178]. Since both pathways are known to play critical roles in cell proliferation and differentiation, several studies demonstrated that SHP2 affects cell proliferation and differentiation in large range of cell types [179–183]. For example, SHP2 is required for the initiation of retinal neurogenesis and it regulates the patterning of optic vesicles by mediating retinal progenitor factors and cell proliferation [184]. Huang and colleagues have shown that the suppression of SHP2 activity reduces cell migration and neurite outgrowth, and that it decreases the differentiation-induced activation of FAK, Src, paxillin, and ERK1/2 [185]. Also, the authors demonstrated that SHP2 is recruited to focal adhesions in NSCs and that it regulates focal adhesion formation [185].

Recent studies have suggested that Shp2 is involved in oligodendrocyte development in the telencephalon. *In vitro* studies using rat cortical cultures demonstrated different roles for Shp2 in either oligodendrocyte precursor cell proliferation or maturation [186, 187]. The in vivo function of Shp2 in oligodendrocyte differentiation was also investigated by Zhu and colleagues using conditional mutant mice with a selective Shp2 deletion in Olig1-expressing cells in the ventral spinal cord [93]. The mutant mice displayed a dramatic reduction in the number of both oligodendrocyte precursor cells and mature oligodendrocytes and decreased axonal myelination in the developing CNS, suggesting that Shp2 is a critical regulator of oligodendrocyte proliferation and differentiation [93]. Similarly, Ehrman and colleagues investigated the role of Shp2 in ventricular zone progenitor cells of the ventral telencephalon and in cells of the oligodendrocyte lineage by deleting Shp2 in *Olig2*-positive cells [94]. Olig2-specific Shp2 null mutant mice showed a significant decrease in the number of oligodendrocyte progenitor cells, at embryonic and postnatal stages, and severe hypomyelination [94]. Moreover, expressing an NS-associated mutation Shp2^Q79R^ using Olig2-Cre increased the number of oligodendrocyte precursor cells in the embryonic and postnatal brain, but also induced abnormal myelination and fewer myelinated axons in the white matter [94].

SHP2 has been shown to play a role in cell fate decisions as it promotes neurogenesis and suppresses astrogliogenesis through the repression of the JAK-STAT pathway, which is required for astrocyte formation in the developing brain. Gauthier and colleagues reported that germline Shp2^D61G^ heterozygous mice showed more neurons and fewer astrocytes in the hippocampus and dorsal cortex at postnatal day 2, and suggested that NS-associated mutations cause brain abnormalities by disrupting the balance of CNS populations [96]. Ke and colleagues also demonstrated that SHP2 is an important player in mammalian brain development by generating a novel mutant mouse in which Shp2 is selectively eliminated in neural precursor cells [92]. The mutant mouse showed early postnatal lethality, decreased proliferation of NSCs, and lamination defects in the developing cerebral cortex [92]. Mutant mice showed a reduced number of neurons and an increased number of astrocytes, which imply defective neuronal differentiation and modestly enhanced astrogliogenesis, supporting the idea that Shp2 promotes neurogenesis and suppresses astrocytogenesis [92]. The peripheral nervous system of Wnt1-Cre or Krox20-Cre conditional Shp2 floxed mice displayed severe deficits in Schwann cell development and the hypomyelination of peripheral nerves [188].

There are other NS mouse models in addition to Shp2 mutant mice. Heterozygous *Raf1*-deficient mice display smaller granule cell layer volumes at postnatal day 30 and a substantial number of abnormal, chromophilic, fast dividing cells in the subgranular zone and dentate gyrus [105]. In addition, *Raf1*-deficient neural progenitor cells showed an increased rate of cell death and reduced neuronal maturation [105]. Recently, Holter and colleagues reported that mice expressing the NS-associated gain of function mutation *Raf1*^L613V^ have a significantly greater density of GFAP-positive astrocytes in the cortex and hippocampus. In addition, the number of Olig-positive oligodendrocyte progenitor cells were also increased in cortical area of *Raf1*^L613V^ mutant mice [107]. Interestingly, *Raf1*^L613V^ mice showed enhanced performance in several learning tasks [107]. NS-associated *Kras*^G12V^ mutant mice showed enhanced GABAergic synaptogenesis and impaired spatial learning when the mutation was selectively expressed in synapses [99].

Although it is known that transcription factors for glial lineage become highly expressed in accordance with increasing RAS-ERK activity [85, 90, 108], RAS-activating mutation SHP2^D61G^ promotes neuronal lineage rather than glial lineage, by direct interaction with JAK-STAT pathway [96]; however, the expression of glial transcription factors that may have been affected by the increase in RAS-ERK activity is yet to be examined. On the contrary, other NS-linked mutations such as *Raf1*^L613V^ rather enhanced glial lineage [107]. Although the underlying mechanism for the discrepancy in cellular phenotypes is not clear, these results suggest that there are distinct pathophysiology according to each NS-associated mutation. It would be interesting to examine the neuron-glia ratio in either NS patient-derived iPSCs or postmortem brain tissues harboring specific *PTPN11* or *RAF1* mutations.

#### Cardio-facio-cutaneous syndrome

Cardio-facio-cutaneous syndrome (CFCS) is a rare RASopathy that is caused by mutations in the genes that encode downstream effectors of RAS [41, 42, 44], including *BRAF* [41, 42], *KRAS* [41], and *MEK1/2* [42]. Importantly, heterozygous *BRAF* mutations are found in over 70% of CFCS patients [58]. *BRAF* encodes a serine/threonine kinase, and, interestingly, both the kinase-active and kinase-impaired mutations of *BRAF* are associated with CFCS [41, 42]. Heterozygous missense mutations in *MEK1* and *MEK2* are found in approximately 25% of CFCS individuals [58]. *MEK1* and *MEK2* are threonine/tyrosine kinases, and all the *MEK* mutants associated with CFCS are activating mutations [42, 189]. CFCS patients display multiple congenital abnormalities which overlap with those seen in NS and Costello syndrome, including craniofacial defects, hypertrophic cardiomyopathy, pulmonary artery stenosis, and neurocognitive delay [58]. CFCS individuals exhibit NS-like faces, with macrocephaly, low-set ears, a short nose, a broad forehead, and down-slanting palpebral fissures with ptosis [4, 59]. Cardiac abnormalities are also similar to those of NS and Costello syndrome, with pulmonic stenosis, septal defects, and hypertrophic cardiomyopathy (HCM) having the highest prevalence [59]. Neurological abnormalities, including hypotonia, motor delay, seizures, tactile defensiveness, speech delay, and learning disabilities, are present at varying degrees [4, 50, 55]. Failure to thrive caused by gastrointestinal dysfunction, including vomiting, oral aversion, reflux, and constipation, is also typical in CFCS individuals in infancy [50]. However, CNS abnormalities are significant diagnostic features of CFCS. Previous studies reported the abnormalities in brain structures, including ventriculomegaly and hydrocephalus, in CFCS patients [44–50]. Volume loss in the brain due to cortical atrophy, cerebral atrophy, brain stem atrophy, and white matter atrophy have also emerged in a subpopulation of patients [44, 46, 51–54]. Additionally, migration abnormalities, myelination abnormalities, and corpus callosum abnormalities, such as hypoplasia and lipoma were also revealed by brain imaging [50, 52, 55–57]. In line with brain abnormalities, most CFCS patients are diagnosed with varying degrees of cognitive deficits and intellectual disabilities [50].

Recently, patient-derived induced pluripotent stem cells (iPSCs) have contributed to advancements in the understanding of disease-associated mutations. Yeh and colleagues generated iPSC from a patient harboring *BRAF*^Q257R^, the most frequent CFCS mutation [190]. This mutation resulted in a depletion of neural progenitor pool, induced by decreased phosphorylation of AKT, and early neuronal maturation [190]. Due to the depletion of progenitors, the number of late-born cells, such as the upper-layer cortical neurons and glia, was decreased [191]. The number of GABAergic interneurons was increased, indicating that the high prevalence of seizures in CFCS individuals may be caused by an imbalance between excitation and inhibition [191].

Fewer animal models of CFCS have been reported likely due to its lower prevalence (1 in 810,000) compared to other RASopathies. Transgenic mouse models carrying gain of function mutations that are associated with CFCS recapitulate multiple aspects of human CFCS patients [108, 192]. Since *BRAF* is the most prevalent gene that is mutated in CFCS, a majority of animal studies in CFCS have focused on *Braf*. Prior to the review of gain of function mouse models associated with CFCS, we first reviewed loss of function studies that investigated the role of *Braf* in various biological processes. Wiese and colleagues identified that cultured embryonic sensory and motor neurons lacking *Braf* could not survive in the presence of neurotrophic factors while *Raf1*-deficient neurons could survive, suggesting that *Braf* is essential for survival [193]. A *Braf* null mutant mouse was embryonically lethal due to the vascular defects at midgestation [194]. In addition, the ablation of *Braf* in NSCs using Nestin-Cre resulted in abnormal morphogenesis of the CNS, such as a decreased cerebellum with fuzzy granule cell layer borders and a diminished hippocampus granule cell layer, due to reduced differentiation of dentate gyrus progenitor cells into mature granule cell neurons [102]. Nestin-Cre specific *Braf*-deficient mice also displayed severe dysmyelination and defective oligodendrocyte differentiation, implicating *Braf* in postnatal CNS development [103]. Forebrain excitatory neuron-specific *Braf* knockout mice showed deficits in hippocampal long-term potentiation and impaired hippocampal-dependent learning and memory, while the impact of *Braf* deletion on CNS development in this knockout remains to be investigated [195].

The first mutant mouse model of CFCS was a knock-in of the constitutively active form of *Braf*, *Braf*^V600E^, which recapitulates several CFCS characteristics, including a reduced life span, growth retardation, facial dysmorphia, cardiomegaly, and epileptic seizures [104]. Mice expressing the conditional knock-in *Braf*^L597V^ mutation also recapitulated CFCS symptoms of a short stature, facial dysmorphia, and cardiac enlargement [196]. The most prevalent CFCS mutation, *Braf*^Q241R^, induced embryonic/neonatal lethality with multiple congenital defects that included embryonic skeletal abnormalities, lymphatic defects, cardiac defects, and liver necrosis in the C57BL/6J background, and lethality between birth and 24 weeks, growth retardation, sparse and ruffled fur, liver necrosis, and atrial septal defects on the mixed background (BALB/c and C57BL/6J) [192, 197]. In addition, *Braf*^Q241R/-^ mice showed growth retardation, a hunched appearance, craniofacial dysmorphism, and learning deficits on ICR background [192].

Mouse models carrying *Mek1*^Y130C^, the most common *MEK1* mutation in CFCS patients, showed increased ERK activation in response to growth factors, pulmonary artery stenosis, cranial dysmorphia, and neurological anomalies [108]. Moreover, *Mek1*^Y130C/Y130C^ mice showed a higher density of GFAP-positive astrocytes in the sensory cortex and hippocampal CA1 regions [108]. In addition, the total cortical oligodendrocyte population, as analyzed by Olig2 immunolabeling, was increased in the sensory cortex of *Mek1*^Y130C/Y130C^ mice [108]. As addressed earlier, patients-derived iPSC containing *BRAF*^Q257R^ exhibited early neuronal maturation and decreased late-born glial populations, whereas either CFCS-associated *Mek1*^Y130C^ or *Braf*^V600E^ expressing adult mice exhibited an increased number of GFAP-positive cells in hippocampal and cortical areas [104, 108, 190]. Although, *BRAF*^Q257R^ is a gain of function mutation, the activation of ERK was decreased in neural progenitor cells, which might have been due to cell context-dependent role of *BRAF*, and these results indicate that the decreased ERK activation may be responsible for the decreased glia in *BRAF*^Q257R^ iPSCs [190]. In addition to mouse models, zebrafish models expressing CFCS *Braf* or *Mek* variants were also generated, and these CFCS mutant alleles interfered with convergence-extension cell movements during gastrulation to cause similar developmental phenotypes [189]. Taken together, each of the CFCS-associated genes play essential roles in CNS development, including oligodendrocyte precursor maturation and proliferation, myelination, and neuronal differentiation. However, our knowledge regarding the causal relationship between CNS abnormalities and cognition in CFCS is still limited. Further studies using mutant animals with more specific temporal and spatial manipulation of CFCS genes would provide understanding of the pathophysiology of cognitive deficits in CFCS.

#### Costello syndrome

Costello syndrome (CS) is a rare multiple congenital abnormality syndrome that affects 1 in 1,250,000 people and shares many features with other RASopathies [198–200]. CS is mostly caused by gain of function mutations in the *HRAS* gene, most of which have been previously reported as somatic or oncogenic mutations in various tumors [60, 62, 201]. *HRAS* activating mutations are highly prevalent in CS individuals; they disrupt guanine nucleotide binding and induce a decrease in intrinsic and GAP-induced GTPase activity, allowing mutant HRAS proteins to remain in the active state [202]. In addition, *BRAF*, *KRAS*, and *MEK1* mutations are also associated with a small population of CS individuals [27, 63, 203]. CS patients are typical characterized by coarse facial features, redundant and loose skin, severe failure to thrive, mental retardation, cardiomyopathy, and a predisposition to tumors [59, 60]. There is no single feature that is unique to CS, and this syndrome phenotypically overlaps with NF1, NS, NSML, and CFCS [60, 204]. Typical and coarse facial features associated with CS involve macrocephaly with a prominent forehead, a short nose with a depressed nasal bridge and a broad base, and low-set, posteriorly rotated ears with thickened helices and lobes. Most CS patients have cardiac abnormalities, including hypertrophic cardiomyopathy, valve abnormalities, septal defects, and arrhythmia [205]. Failure to thrive due to gastrointestinal dysfunction often involves reflux, oral aversion, and constipation during early infancy [67, 200]. Structural and electrophysiological neurological malformations are also common in CS. For example, ventricular abnormalities, such as mild ventricular dilatation, are observed in more than 40% of CS individuals [64–67]. Cerebral malformations in CS include cerebral atrophy, leukomalacia, poor gray-white matter differentiation, a small corpus callosum, and MRI signal abnormalities [64, 65, 67–71]. Cerebellar abnormalities include malformation, cerebellar atrophy, deviation of the cerebellar tonsils, and demyelinization of the basal tonsil [66, 69, 71–74].

Krencik and colleagues have shown that human iPSCs carrying *HRAS*^G12S^ that were derived from CS patients exhibited hyperplasia and differentiated into astroglia more rapidly in vitro than iPSCs derived from control cell lines with normal *HRAS*. CS-derived iPSCs also generated an abundance of extracellular matrix remodeling factors and proteoglycans [206]. Moreover, *HRAS*^G12S^ iPSC-derived neurons had a longer progenitor phase, unlike the phenotype reported in *BRAF*^Q257R^ iPSC-derived neurons that originated from CFCS patients [190, 207]. Thus, postnatal progressive cerebellar overgrowth of the brain in CS individuals could be caused by the extended progenitor phase [208].

As with CFCS, only few animal models were generated for CS. Both homozygous and heterozygous *Hras*^G12V^ knock-in mice closely phenocopied some of the features observed in individuals with CS, including facial dysmorphia, cardiomyopathies, and alterations to the homeostasis of the cardiovascular system [209]. In addition, later studies with homozygous *Hras*^G12V^ knock-in mice demonstrated that they have neurocognitive deficits, such as hyperactivity, increased anxiety-like behavior and mild deficit in spatial memory [210]. However, Viosca and colleagues did not observe significant changes in either the activity or the expression of downstream of *Hras* such as phospho-CREB and *c-fos* [210]. Transgenic mice with forebrain excitatory neuron-specific expression of *Hras*^G12V^ under the control of the αCaMKII promoter displayed several synaptic phenotypes, including a high density of docked neurotransmitter vesicles in glutamatergic terminals and increased synaptic plasticity which may be associated with the dramatically enhanced hippocampal-dependent learning [101]. Schreiber and colleagues have also shown that the homozygous *Hras*^G12V^ knock-in mice exhibit spatial learning deficits, which are accompanied by robust upregulation of Erk signaling in hippocampal lysates, neuronal hypertrophy, increased brain volume, and impaired mGluR-dependent long-term depression (LTD) [100]. Notably, mice expressing CS-associated *Hras*^G12V^ or *Hras*^G12S^ mutations in cortical precursors displayed promoted precursor cell proliferation and premature gliogenesis, but inhibited neurogenesis [211]. Consistently, either form of *Hras* mutations also promoted precursor cell proliferation and astrogenesis, but inhibited neurogenesis in cultured cortical precursors [211]. These findings from multiple experimental systems such as iPSCs, mice models and cultured cells commonly suggest the essential role of *HRAS* in neural precursor cell proliferation and gliogenesis, which might strongly affect the structure and function of CNS including increased brain volume in CS patients.

## Concluding remarks

**Fig. 2:**
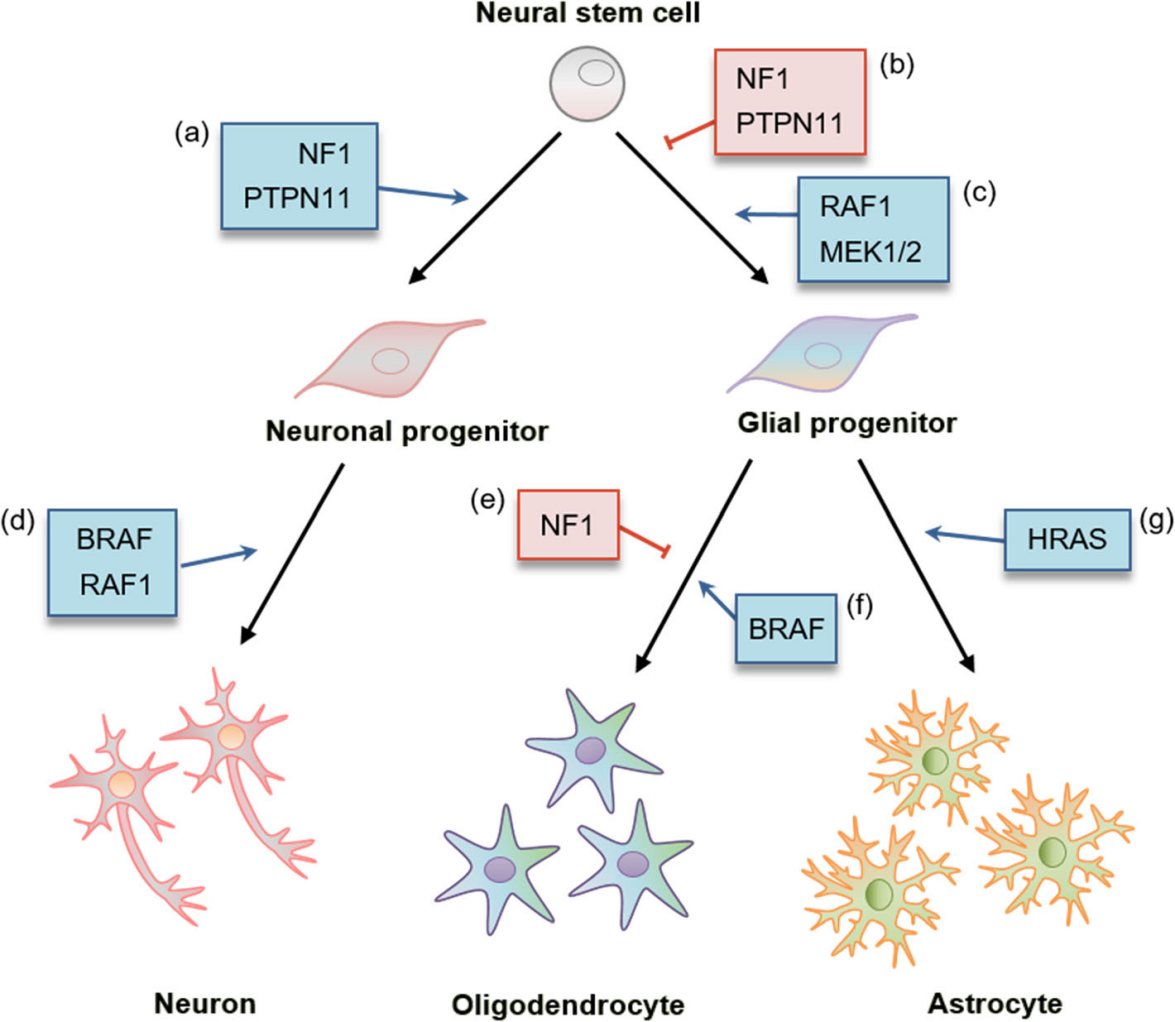
Effect of RAS signaling components on neural stem cell differentiation. Neural stem cells are able to generate progeny cells that terminally differentiate into neurons, oligodendrocytes, and astrocytes. **a** NF1 inactivation led to decreased neurogenesis in neonatal and adult mouse brains [85]. PTPN11 positively regulates neurogenesis at the expense of gliogenesis [96]. **b** NF1 negatively regulates gliogenesis, thus NF1 inactivation increases the number of glial progenitor cells and gliogenesis [78, 79, 85, 89, 159, 160]. PTPN11 suppresses gliogenesis by directly interacting with the JAK-STAT pathway, which promotes gliogenesis [92, 96]. **c** Hyperactivation of RAF1 induces the increase of glial lineage populations, including oligodendrocyte progenitor cells and astrocytes [107]. MEK is required for gliogenesis, and the hyperfunction of MEK1 leads to increase in glial populations [108, 109]. **d** BRAF and RAF1 positively regulate neuronal differentiation, and the disruption of BRAF or RAF1 impairs the ability of progenitor cells to differentiate into mature neurons in mouse brain [102, 105]. In consistent, iPSC containing hyperactivated BRAF mutant showed early maturation of neurons [190]. **e** Oligodendroglial lineage potential is restricted by NF1 in the adult hippocampus, and inactivation of NF1 allows the adult hippocampus to generate oligodendrocytes [85]. **f** BRAF is required for oligodendrocyte maturation and myelination during postnatal development [103]. **g** Hyperactivated HRAS leads to an acceleration of astroglial maturation [206, 211]. Blue and red arrows indicate positive and negative regulation, respectively.

Here, we reviewed that mutations in different components of the RAS-ERK signaling pathway associated with different RASopathies have distinct impacts on CNS development in a cell type-specific manner (Fig. 2). However, it is still unclear how some mutations affect neurons and others affect glia. One hypothesis is that different signaling molecules are expressed at distinct phases during development. So far, we do not have the expression profiles of RAS signaling molecules during brain development in high spatial and temporal resolution. However, most of the key RAS-ERK components, such as NF1, SHP2, BRAF, and MEK1/2, begin to be expressed before either embryonic day 10 or 15, which are initiation time points of neurogenesis or astrogliogenesis, respectively [212]. Recently, it has been shown that the expression of RAS-ERK signaling molecules was remarkably different between excitatory and inhibitory neurons in mouse hippocampus [162]. Thus, it would be interesting to examine whether the expression levels of various signaling molecules are differentially regulated in NSCs over different developmental stages. Advanced tools, such as single cell RNA-seq, might be useful to answer this question. It should also be considered that there are multiple cross-talks between RAS and other signaling pathways. Different components in RAS signaling interact with distinct signaling networks, which may account for the cell type-specific developmental deficits in each RASopathy.

Treatments for the cognitive deficits found in RASopathies are not available yet. Since most RASopathy-associated mutations increase RAS-ERK activation, downregulating the activity of RAS or its downstream effectors is an obvious strategy to develop treatments for RASopathies. Although statins, which can reduce RAS activity by inhibiting the farnesylation of RAS, have been proposed for the improvement of learning disabilities in NF1 children [213], the results from various clinical trials have been inconsistent [214–216]. The reason for these discrepant results remains unclear. However, considering the ubiquitous expression of RAS in many cell types and multiple organs, directly regulating RAS activity may have unknown confounding effects. Thus, it would be better to target specific molecules other than RAS in a disease-specific manner. For example, Omrani and colleagues showed that inhibitory neuron-specific attenuation of hyperpolarization-activated cyclic nucleotide-gated (HCN) currents can be an underlying mechanism for the cognitive deficits in *Nf1*^+/-^ mice when they used an HCN agonist to rescue cognitive deficits in *Nf1*^+/-^ mice [217]. Recently, Ryu and colleagues showed that selectively reducing the interaction between mutant SHP2 and Gab1 in excitatory neurons reversed the physiological and behavioral deficits in a mouse model of NS [162]. Conditional mutant mice with higher spatial and temporal resolution will provide clues when, where, and which cell types are most suited for interventions.

Lastly, it should be noted that most of the RASopathy mechanism studies have used mice as a model system. Mouse models have many advantages and can be used to study neuropsychiatric disorders because the majority of neuropsychiatric drugs used in humans were shown to be, at least partially, effective in mouse models [218]. However, caution is still warranted. Nowadays, it has become relatively easy to model diseases in vitro using iPSC and several iPSC lines are available to study RASopathies, allowing for parallel and comparative analyses in vitro and in vivo.

## Data Availability

Not applicable

## Abbreviations

Ascl1: Achaete scute-like 1
BLBP: Brain lipid binding protein
CFCS: Cardio-facio-cutaneous syndrome
CKO: Conditional knockout
CNS: Central nervous system
CS: Costello syndrome
ERK: Extracellular signal-regulated kinase
GABAergic: Gamma-aminobutyric acidergic
GAPs: GTPase activating proteins
GEFs: Guanine nucleotide exchange factors
GFAP: Glial fibrillary acidic protein
GNPs: Granule neuron progenitors
HCM: Hypertrophic cardiomyopathy
iPSCs: Induced pluripotent stem cells
JAK: Janus kinase
KRAS: Kirsten rat sarcoma viral oncogene homolog
MEK1/2: MAPK/ERK kinase 1/2
Neurog2: Neurogenin 2
NF1: Neurofibromatosis type 1
NRAS: Neuroblastoma RAS viral oncogene homolog
NS: Noonan syndrome
NSCs: Neural stem cells
NSML: Noonan syndrome with multiple lentigines
PI3K: Phosphatidylinositol 3-kinase
PTPN11: Protein tyrosine phosphatase non-receptor type 11
RTKs: Receptor tyrosine kinases
SHOC2: Soc-2 suppressor of clear homolog
SOS1: Son of sevenless homolog 1
STAT: Signal transducer and activator of transcription
